# Characterization of *ESR1* alterations in patients with breast and gynecologic cancers

**DOI:** 10.1186/s13058-025-02217-0

**Published:** 2026-01-19

**Authors:** Gargi D. Basu, Paige E. Innis, Angela K. Deem, Arthur Starodynov, Sameer S. Udhane, Szabolcs Szelinger, Min Wang, Janine R. LoBello, Frederick L. Baehner, Jean-Paul De La O, Joyce O’Shaughnessy

**Affiliations:** 1 Exact Sciences Corporation, 5505 Endeavor Ln, Madison, WI 53719 USA; 2 Baylor University Medical Center, Texas Oncology, Sarah Cannon Research Institute, 3410 Worth Street, Suite 400, Dallas, TX 75246 USA

**Keywords:** Estrogen receptor, Resistance, Breast cancer, Gynecologic cancers, Comprehensive genomic profiling

## Abstract

**Background:**

*ESR1* alterations present a common mechanism of resistance to endocrine therapy (ET) in hormonally driven tumors. The clinical significance of these alterations continues to evolve with newly approved targeted therapies and a range of ongoing investigational trials.

**Methods:**

A retrospective study of 2574 breast cancer (BC) and 1110 gynecologic cancer samples that underwent whole exome and whole transcriptome profiling was conducted to assess the distribution of *ESR1* and associated co-alterations in local (primary breast or regional lymph node) versus metastatic BC samples and in the major BC subtypes. Prior treatment history was unknown.

**Results:**

*ESR1* alterations were present in 6.2% (n = 159/2574) of BC samples and 3.4% (n = 38/1110) of gynecologic cancer samples. In HR + /HER2- BC, *ESR1* alterations overall and *ESR1* missense mutations were more frequent in samples from metastatic compared to local/regional sites (overall: n = 86/321 (26.8%) and n = 53/1427 (3.7%), respectively (*P* < 0.001); missense: n = 72/321 (22.4%) and n = 20/1427 (1.4%), respectively (*P* < 0.001)). Whole transcriptome sequencing detected *ESR1* fusion genes in 2.1% (n = 55/2574) of BC samples and in 1.9% (n = 21/1110) of gynecologic cancer samples, and *CCDC170* was the most common fusion partner in both cancer types. In HR + /HER2- BC, *ESR1* fusions were more common in metastatic samples compared to local/regional (n = 17/321 (5.3%) and n = 29/1427 (2.0%), respectively; *P* < 0.001). Evaluation of 21 therapeutically actionable biomarkers identified co-alterations enriched in *ESR1*-altered HR + /HER2- BC, including *FGF3/4/19* and *CCND1* amplifications. No significant co-alterations were found in gynecologic cancer samples.

**Conclusions:**

*ESR1* alterations were most frequent in HR + /HER2- BC samples and missense mutations were more frequent in metastatic samples, consistent with their role in ET resistance and disease progression. *ESR1* alterations co-occurred with therapeutically relevant alterations in other genes that may help inform clinical decision-making. Gynecologic tumors harbored *ESR1* alterations that have prognostic and potentially therapeutic relevance.

**Supplementary Information:**

The online version contains supplementary material available at 10.1186/s13058-025-02217-0.

## Background

The estrogen receptor α (ER) is a ligand-dependent transcription factor encoded by the *ESR1* gene. The ER-ligand complex regulates numerous cellular activities, including differentiation, proliferation, and survival by binding specific DNA sequences called estrogen response elements to regulate expression of estrogen-responsive genes. Estrogen receptor α can also bind the plasma membrane, where it interacts with PI3K and activates PI3K/AKT/mTOR signaling. Dysregulated ER activity can result in pathological processes that promote tumorigenesis, progression, and invasion as well as affect the response to endocrine therapy (ET) in patients with hormonally dependent cancers.

Almost 70% of all breast cancer (BC) cases diagnosed in the United States are hormone receptor positive (HR +) [1], which are defined as tumors in which at least 1% of tumor cells express either ER or progesterone receptor [2]. Estrogen receptor-positive BC is the most common molecular subtype in early as well as advanced disease [3], and the therapeutic landscape for these cancers is expanding with new classes of drugs for patients with de novo and recurrent BC. Endocrine therapy, which involves either estrogen deprivation using aromatase inhibitors (AIs) or ER modulation with the selective estrogen receptor modulator (SERM) tamoxifen, is commonly prescribed as an adjuvant treatment to women with HR + BC. Recently, the addition of CDK4/6 inhibitors to ET as first-line therapy for metastatic disease has improved overall survival (OS) and progression-free survival [4–7]; however, nearly all patients eventually develop disease progression through various mechanisms of endocrine resistance [8].

*ESR1* alterations are recognized as a key mechanism of resistance to ET in HR + advanced and metastatic BC [9–11], and these events have been characterized broadly in BC patients [12]. Multiple categories of genomic aberrations have been documented including missense mutations, genomic rearrangements (including gene fusions), and copy number variations. *ESR1* alterations are associated with more aggressive biology and poorer prognosis [13] and are mainly detected by circulating tumor DNA (ctDNA) in patients with metastatic disease [12]. In HR + /HER2- BC, *ESR1* alterations are most frequently acquired after first-line AI therapy [14–16], resulting in AI resistance and necessitating a change in treatment. Screening for *ESR1* alterations, which drive ligand-independent activation of ER, is now standard of care for patients with HR + /HER2- metastatic disease that has progressed on adjuvant or metastatic ET [17].

Second-line targeted therapies have been developed to combat ET resistance mechanisms, including *ESR1* alterations. For instance, selective estrogen receptor degraders (SERDs), which are competitive ER agonists that target the receptor for proteasome-dependent degradation, have been developed to overcome *ESR1*-mediated resistance [18, 19]. Fulvestrant was the first SERD approved by US FDA for the treatment of HR + /HER2- metastatic BC [20]. Although some *ESR1*-altered HR + /HER2- BCs may remain responsive to fulvestrant, it has limited bioavailability and must be administered in monthly intramuscular injections [21]. In January 2023, elacestrant was approved as an orally available SERD for the treatment of ER + /HER2- *ESR1*-mutated advanced or metastatic BC that has progressed after ET [22], making this therapy more accessible for patients with AI resistance. There are multiple ongoing clinical trials evaluating the effectiveness of next-generation SERDs in this patient population [23]. For patients with disease that has progressed on ET and that harbor activating mutations in the PI3K/AKT pathway (including *AKT* and *PIK3CA* mutations or *PTEN* loss-of-function alterations), the FDA has approved use of either capivasertib or alpelisib plus fulvestrant [24, 25] or inavolisib in combination with palbociclib and fulvestrant [26].

Certain gynecologic cancers also commonly express ER, and ET can be considered as treatment for advanced cancers, in particular, ovarian and low-grade endometrial cancers. However, the landscape of *ESR1* alterations in gynecologic cancers has not been widely reported. In one large cohort of gynecologic cancer samples, 3% had an *ESR1* alteration [27], which were enriched in carcinomas with endometrioid histology. In another study, an *ESR1* hotspot mutation was detected in 15% (9/60) of primary ovarian cancer tissues [28]. Although less frequent compared to advanced BC, detection of *ESR1* alterations in gynecologic tumors provides important information for clinical decision-making [27].

The clinical significance of *ESR1* alterations continues to evolve with newly approved targeted therapies and a widening landscape of ongoing investigational trials. Here, we characterize *ESR1* alterations in patients with breast or gynecologic cancer that underwent whole exome and whole transcriptome profiling. We also evaluate the clinical relevance of co-occurring alterations in *ESR1*-altered breast and gynecologic cancers.

## Materials and methods

### Patient population

This was a retrospective study of 3684 samples from patients with breast or gynecologic solid tumors obtained between April 2018 and March 2024 and analyzed with the OncoExTra® assay, formerly known as the GEM ExTra® assay (Exact Sciences, Phoenix, Arizona, USA). Patients with more than one BC subtype listed on different reports, presumably due to subtype switching, were excluded. All patient data were deidentified prior to inclusion, and the study was approved under IRB 20–001 (approval #201,818,630).

### Tumor genomic sequencing

Matched-normal whole exome sequencing and whole transcriptome sequencing were performed on tumor and blood samples obtained as part of routine clinical care in a College of American Pathologists (CAP)-accredited, Clinical Laboratory Improvement Amendments (CLIA)-certified laboratory using the OncoExTra comprehensive genome profiling assay methodology, which was previously published [29]. Breast and gynecologic tumor tissue was evaluated by a pathologist and when necessary macrodissection was performed to ensure that samples contained > 20% tumor cells and < 60% necrotic tissue. Briefly, DNA from all samples was extracted and sheared using commercially available kits (Qiagen). Targeted sequences from prepared DNA and RNA libraries (Roche) were captured using a custom oncology-specific probe set (Integrated DNA Technologies). The captured regions were amplified, and the DNA and RNA samples were pooled and sequenced (Illumina). Sequencing data were processed using a custom analysis pipeline. The DNA workflow of the OncoExTra assay reports on single nucleotide variants (SNVs), indels, copy number amplifications and deletions, tumor mutational burden (TMB), and microsatellite instability (MSI). The RNA workflow reports on gene fusions and on five alternative transcripts: *ARv7*, *METe14*, *EGFRvIII*, and *EGFRvIVa/b*.

### Identification of co-occurring alterations

Unless otherwise indicated, for analyses of co-alterations, genes were included if they were present in at least 2.5% of *ESR1*-altered or *ESR1*-non-altered samples and the alterations were considered actionable. Actionable alterations were defined as somatic alterations with documented response to US FDA-approved drugs (including in the cancer type with the approval (on-label) or in a different cancer type (off-label)), with investigational agents available in matched clinical trials, or with evidence of response in cancer guidelines or the literature with possible matched therapies. For the analysis of co-altered genes that confer resistance to ET, the following genes were included: *BRAF*, *CTCF*, *EGFR*, *ERBB2*, *ERBB3*, *FOXA1, HRAS*, *KRAS*, *MAP2K1*, *MYC*, *NF1*, *RB1*, *TBX3,* and *TP53* [10, 30]*.*

### Data interpretation and statistical analyses

All analyses were performed separately for breast and gynecologic cancers. Specimen sites were grouped into local/regional, metastatic, or undefined (gynecologic only) categories. Local/regional BC specimen sites included breast, axillary and other regional lymph nodes, with all other sites grouped as metastatic. Local/regional gynecologic cancer sites were defined based on reported tumor type and specimen site. Tumor specimens that could not be categorized to either local/regional or metastatic were called ‘Undefined’. BCs were evaluated both overall and by breast cancer subtype (HR + /HER2-, HER2 + , triple negative BC (TNBC), and BC not otherwise specified (NOS)). In BC overall and in each BC subtype, metastatic sites of disease that represented < 5% of total metastatic samples or fewer than 2 samples were categorized as ‘Other’. Gynecologic cancer types were evaluated overall and by gynecologic cancer type, including ovarian, endometrial, and cervical cancers; less common gynecologic cancers (carcinoma of fallopian tube, malignant tumor of female genital organ, malignant tumor of vulva, squamous cell carcinoma of vagina) were analyzed together as ‘Other’ gynecologic tumors. For gynecologic cancer samples, metastatic sites of disease that represented < 5% of the total metastatic samples were categorized as ‘Other’.

Descriptive statistics were used to summarize samples by tumor type, BC subtype, age, specimen site (local/regional or metastatic), metastatic site, and *ESR1* alteration status (yes/no). The distribution of sample characteristics by *ESR1* alteration status was evaluated using Chi-Square or Fisher’s Exact tests. Pairwise comparisons were adjusted using Bonferroni correction. Variant allele frequency (VAF) by specimen site was evaluated using the Wilcoxon Rank-Sum test. Co-alteration analysis was performed using Fisher’s Exact test, with Benjamini–Hochberg correction for false discovery rate (FDR), where applicable. SAS 8.4 software was used to perform all analyses and R Version 4.4.0, RStudio 2024.01.1 Build 748 and cBioPortal were used to generate figures. The level of statistical significance was set at *P* < 0.05.

## Results

### Study population

A total of 2574 BC and 1110 gynecologic cancer samples were included in this study (Table 1). Most of the BC samples were from HR + /HER2- tumors (n = 1748 (67.9%)), with smaller proportions of HER2 + (n = 369 (14.3%)), triple-negative BC (TNBC; n = 387 (15.0%)), and not otherwise specified (NOS; n = 70 (2.7%)) samples. Among gynecologic cancer samples, ovarian cancer (n = 475 (42.8%)) and endometrial cancer (n = 461 (41.5%)) were most prevalent, while 102 samples (9.2%) and 72 samples (6.5%) were from cervical cancer and other gynecologic cancers, respectively. Most BC samples were obtained from a local/regional tumor site (n = 2102 (81.7%)). In contrast, approximately half of gynecologic samples (n = 572 (51.5%)) were excised from a local/regional tumor site, and 40.8% (n = 453) were from metastatic sites (site was unknown for 85 samples (7.7%)). For both BC and gynecologic cancer, most samples were obtained from patients ≥ 50 years old (n = 1869 (72.6%) and n = 890 (80.2%), respectively).

**Table 1:** Tumor sample characteristics in BC and gynecologic cancer patients, overall and by *ESR1* alteration status

Characteristics		Overall	*ESR1* alteration	No *ESR1* alteration	*P*-value^a^
All BC samples		2574	159 (6.2%)	2415 (93.8%)	
Receptor subtype	HER2 +	369 (14.3%)	14 (3.8%)	355 (96.2%)	< 0.001
	HR + /HER2-	1748 (67.9%)	139 (8.0%)	1609 (92.0%)	
	TNBC	387 (15.0%)	1 (0.3%)	386 (99.7%)	
	NOS	70 (2.7%)	5 (7.1%)	65 (92.9%)	
Age (years)	< 50	702 (27.3%)	23 (3.3%)	679 (96.7%)	< 0.001
	≥ 50	1869 (72.6%)	135 (7.2%)	1734 (92.8%)	
	Missing	3 (0.1%)	1 (33.3%)	2 (66.7%)	
Specimen site^b^	Local/regional	2102 (81.7%)	65 (3.1%)	2037 (96.9%)	< 0.001
	Metastatic	472 (18.3%)	94 (19.9%)	378 (80.1%)	
Metastatic site^c^	Liver	151 (32.0%)	48 (31.8%)	103 (68.2%)	< 0.001
	Bone	53 (11.2%)	17 (32.1%)	36 (67.9%)	
	Skin	55 (11.7%)	5 (9.1%)	50 (90.9%)	
	Chest Wall	41 (8.7%)	4 (9.8%)	37 (90.2%)	
	Lung	37 (7.8%)	2 (5.4%)	35 (94.6%)	
	Brain	24 (5.1%)	3 (12.5%)	21 (87.5%)	
	Soft Tissue	19 (4.0%)	3 (15.8%)	16 (84.2%)	
	Other	92 (19.5%)	12 (13.0%)	80 (87.0%)	
All gyn samples		1110	38 (3.4%)	1072 (96.6%)	
Tumor type	Ovary	475 (42.8%)	12 (2.5%)	463 (97.5%)	0.21
	Endometrial	461 (41.5%)	25 (5.4%)	436 (94.6%)	
	Cervix	102 (9.2%)	0 (0.0%)	102 (100.0%)	
	Other gynecologic^d^	72 (6.5%)	1 (1.4%)	71 (98.6%)	
Age (years)	< 50	216 (19.5%)	3 (1.4%)	213 (98.6%)	0.07
	≥ 50	890 (80.2%)	35 (3.9%)	855 (96.1%)	
	Missing	4 (0.4%)	0 (0.0%)	4 (100.0%)	
Specimen site^e^	Primary	572 (51.5%)	19 (3.3%)	553 (96.7%)	0.80
	Metastatic	453 (40.8%)	15 (3.3%)	438 (96.7%)	
	Undefined	85 (7.7%)	4 (4.7%)	81 (95.3%)	
Metastatic site^f^	Omentum	91 (20.1%)	4 (4.4%)	87 (95.6%)	0.94
	Lymph Node	45 (9.9%)	1 (2.2%)	44 (97.8%)	
	Colon	35 (7.7%)	1 (2.9%)	34 (97.1%)	
	Peritoneum	32 (7.1%)	0 (0.0%)	32 (100.0%)	
	Abdominal Mass	23 (5.1%)	0 (0.0%)	23 (100.0%)	
	Other	227 (50.1%)	9 (4.0%)	218 (96.0%)	

^a^*P*-values were derived from Chi-Square Tests; Comparisons across *ESR1* alterations exclude missing values

^b^Local/regional defined as specimen site including breast/lymph node/axilla; Metastatic defined as any other specimen site

^c^Denominator is number of patients with metastatic site; Metastatic sites that occur in < 5% of metastatic sites and less than 2 samples by BC subtype are categorized as 'Other'

^d^Other gynecologic samples included carcinoma of fallopian tube, malignant tumor of female genital organ, malignant tumor of vulva, squamous cell carcinoma of vagina

^e^Primary defined as reported tumor type and specimen site; Metastatic defined as any other specimen site

^f^Denominator is number of patients with metastatic site; Metastatic sites that occur in < 5% of metastatic samples are categorized as 'Other'

### Characterization of ESR1 alterations in BC

**Fig. 1:**
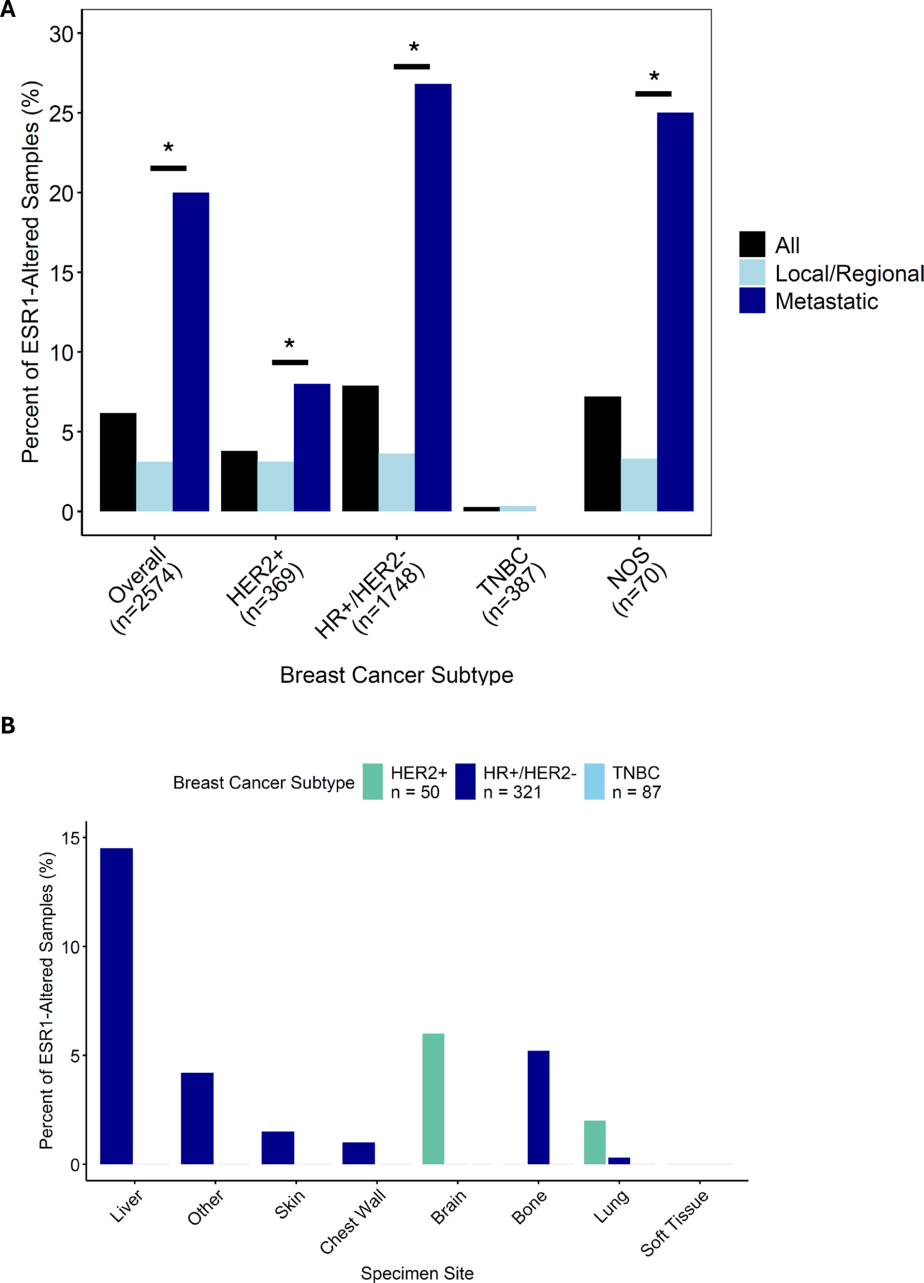
Prevalence and distribution of *ESR1* alterations in BC, overall and by specimen site. A. Prevalence of *ESR1* alterations in local/regional and metastatic BC samples overall and by subtype. B. Distribution of *ESR1*-altered metastatic BC samples by subtype. Statistical comparisons were performed using Chi-Square or Fisher’s Exact Tests. *P < 0.001

Overall, *ESR1* alterations were detected in 6.2% of BC samples (n = 159) and were enriched in HR + /HER2- BC samples (n = 139; 8.0%) (*P* < 0.001; Fig. 1A, Table 1). Samples obtained from patients with BC ≥ 50 years of age were more likely to have an *ESR1* alteration (*P* < 0.001) (Table 1). The frequency of *ESR1* alterations was significantly higher in metastatic BC samples overall and in HR + /HER2- BC samples compared to local/regional samples (overall: n = 94 of 472 (19.9%) versus n = 65 of 2102 (3.1%), respectively; HR + /HER2-: n = 86 of 321 (26.8%) versus n = 53 of 1427 (3.7%), respectively; *P* < 0.001 for both comparisons) (Fig. 1A). No metastatic TNBC samples harbored an *ESR1* alteration. Because we observed a significant difference in the distribution of *ESR1* alterations across metastatic sites (*P* < 0.001) (Table 1), we examined *ESR1* alteration frequency by BC subtype and by metastatic site. The distribution remained significantly different across 139 *ESR1*-altered metastatic and local/regional HR + /HER2- BC samples (*P* < 0.001) as well as in BC NOS, although the latter category only included 5 events (*P* = 0.005) (Supplemental Table 1). In metastatic BC samples, *ESR1* alterations were most prevalent in liver (n = 48 (10.2%) and bone (n = 17 (3.6%)) and least prevalent in skin and lung metastases, and pairwise comparisons uncovered a statistically significant difference between *ESR1* alteration prevalence in both of these tissues compared to metastases in either skin or lung (*P* < 0.05 for all comparisons; Fig. 1B and Supplemental Table 2).

**Table 2:** Actionable co-alterations in HR + /HER2- BC samples, overall and by *ESR1* alteration status

Co-mutated Biomarker	Overall (N = 1748)	*ESR1* alteration (N = 139)	No *ESR1* alteration (N = 1609)	q-value^a^
*FGF3*	169 (9.7%)	30 (21.6%)	139 (8.6%)	< 0.001
*FGF4*	162 (9.3%)	29 (20.9%)	133 (8.3%)	< 0.001
*FGF19*	216 (12.4%)	34 (24.5%)	182 (11.3%)	< 0.001
*CCND1*	254 (14.5%)	33 (23.7%)	221 (13.7%)	0.013
*FGFR1*	125 (7.2%)	17 (12.2%)	108 (6.7%)	0.14
*CDKN2A*	25 (1.4%)	5 (3.6%)	20 (1.2%)	0.15
*PIK3CA*	796 (45.5%)	52 (37.4%)	744 (46.2%)	0.15
*WHSC1L1*	19 (1.1%)	4 (2.9%)	15 (0.9%)	0.15
*PPM1D*	36 (2.1%)	5 (3.6%)	31 (1.9%)	0.44
*NF1*	37 (2.1%)	5 (3.6%)	32 (2.0%)	0.44
*PTEN*	140 (8.0%)	7 (5.0%)	133 (8.3%)	0.48
*ARID1A*	114 (6.5%)	12 (8.6%)	102 (6.3%)	0.50
*AKT1*	88 (5.0%)	4 (2.9%)	84 (5.2%)	0.50
*IGF1R*	44 (2.5%)	5 (3.6%)	39 (2.4%)	0.59
*ERBB2*	52 (3.0%)	2 (1.4%)	50 (3.1%)	0.60
*TP53*	408 (23.3%)	36 (25.9%)	372 (23.1%)	0.61
*MDM2*	49 (2.8%)	5 (3.6%)	44 (2.7%)	0.72
*MAP2K4*	55 (3.1%)	3 (2.2%)	52 (3.2%)	0.72
*SF3B1*	45 (2.6%)	4 (2.9%)	41 (2.5%)	0.86
*BRCA2*	81 (4.6%)	7 (5.0%)	74 (4.6%)	0.87
*MAP3K1*	182 (10.4%)	15 (10.8%)	167 (10.4%)	0.89

Genes included if present in at least 2.5% of *ESR1*-altered or non-altered samples

Sorted by descending significance of co-alteration

^a^Fisher's Exact Test adjusted using Benjamini–Hochberg FDR q-value

**Fig. 2:**
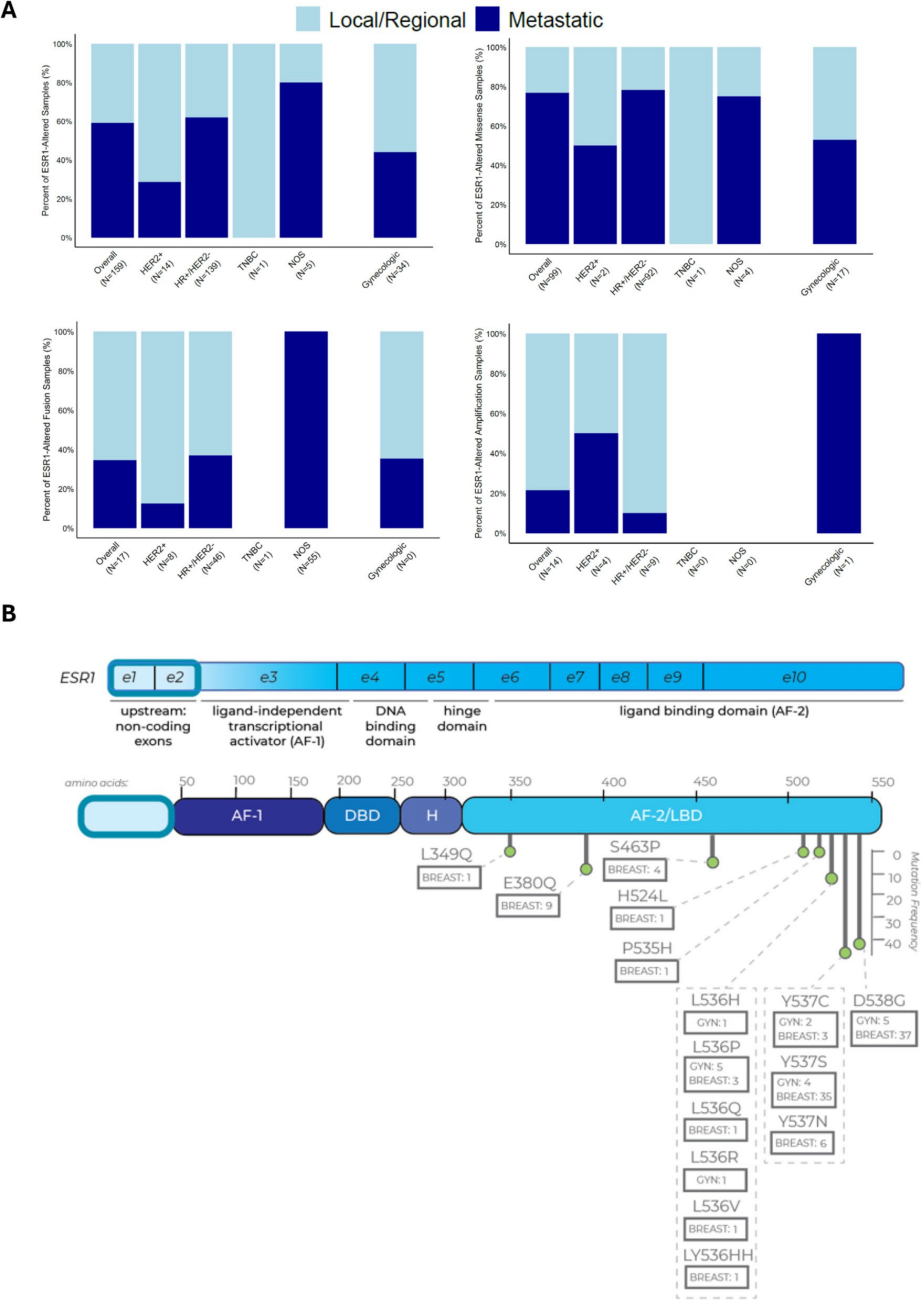
Characterization of *ESR1* alterations in BC and gynecologic cancer samples. A. Distribution of *ESR1* alteration types in BC by subtype and gynecologic cancer samples. Black and white numbers in bars indicate the number of indicated alterations in local/regional or metastatic samples, respectively. B. Distribution of *ESR1* mutations in the LBD in breast and gynecologic cancer patients

**Fig. 3:**
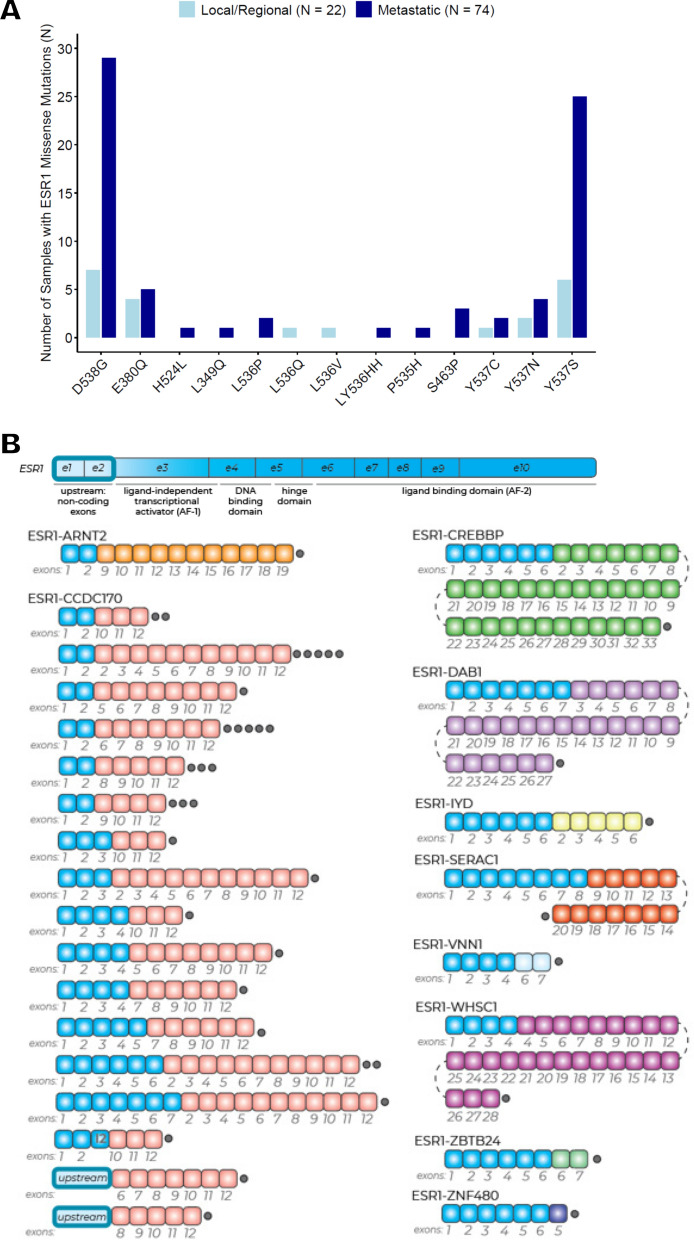
Molecular characteristics of LBD mutations and *ESR1* fusions in HR + /HER2- BC. A. Distribution of *ESR1* missense mutations in HR + /HER2- BC samples. Four samples had polyclonal mutations with more than one *ESR1* mutation present: D538G and L536V, P535H and Y537S, E380Q and S453P, and D538G and Y537S. B. *ESR1* fusions in HR + /HER2- BC samples. Gray dots indicate number of events

We noted differences in the distribution of *ESR1* alteration types among BC subtypes and specimen sites (Fig. 2A). A total of 99 (62.3%) of the 159 samples with *ESR1* alterations across all BC samples had missense mutations, and 92.9% (n = 92) of samples with missense mutations were in HR + /HER2- BC (Fig. 2A, B and Supplemental Table 3). We also observed four samples with polyclonal *ESR1* missense mutations in the HR + /HER2- cohort (Fig. 3A). In TNBC, only one *ESR1* missense mutation in a local/regional sample was observed among 387 samples (0.3%; Fig. 2A, B and Supplemental Table 3). In both overall BC and HR + /HER2- BC samples, missense mutations were significantly more frequent in metastatic samples versus local/regional samples (n = 76 (16.1%) versus n = 23 (1.1%) and n = 72 (22.4%) versus n = 20 (1.4%), respectively; *P* < 0.001 for both comparisons) (Fig. 2A and Supplemental Table 3). In HR + /HER2- BC, where activating mutations in the *ESR1* ligand-binding domain (LBD) can necessitate a change in therapy owing to ET resistance, we observed a concentration of mutations in known hotspots, including D538G (n = 36, 2.1%) and Y537S/N/C (n = 40, 2.3%) mutations (Figs. 2B and 3A). Among HR + /HER2- BC samples with *ESR1* missense mutations, the distribution of median VAF for missense *ESR1* mutations was lower for local/regional compared with metastatic samples (median = 15 vs. 23, respectively; *P* = 0.019) (Supplemental Table 4).

**Table 3:** Co-alterations in HR + /HER2- BC samples that confer resistance to ET, overall and by *ESR1* alteration status

Co-mutated Biomarker	Overall (N = 1748)	*ESR1* (N = 139)	No *ESR1* alteration (N = 1609)	q-value^a^
*BRAF*	1 (0.1%)	1 (0.7%)	0 (0.0%)	0.80
*NF1*	37 (2.1%)	5 (3.6%)	32 (2.0%)	0.84
*KRAS*	23 (1.3%)	0 (0.0%)	23 (1.4%)	0.84
*ERBB2*	52 (3.0%)	2 (1.4%)	50 (3.1%)	0.93
*TP53*	408 (23.3%)	36 (25.9%)	372 (23.1%)	0.93
*RB1*	27 (1.5%)	1 (0.7%)	26 (1.6%)	1.00
*MYC*	33 (1.9%)	2 (1.4%)	31 (1.9%)	1.00
*ERBB3*	16 (0.9%)	1 (0.7%)	15 (0.9%)	1.00
*EGFR*	8 (0.5%)	0 (0.0%)	8 (0.5%)	1.00
*HRAS*	1 (0.1%)	0 (0.0%)	1 (0.1%)	1.00

^a^Fisher's Exact Test adjusted using Benjamini–Hochberg FDR q-value

Sorted by descending significance of co-alteration

**Table 4:** *PIK3CA* alterations in HR + /HER2- BC samples with *ESR1* missense mutations, overall and by specimen site

*PIK3CA* mutation^a^	All Samples^b^ (N = 92) n (%)	Local/Regional (N = 20) n (%)	Metastatic (N = 72) n (%)	P-value^c^
Any *PIK3CA* Mutation	35 (38.0%)	7 (35.0%)	28 (38.9%)	0.49
*PIK3CA* (H1047R)	17 (18.5%)	3 (15.0%)	14 (19.4%)	
*PIK3CA* (E542K)	6 (6.5%)	1 (5.0%)	5 (6.9%)	
*PIK3CA* (N345K)	5 (5.4%)	1 (5.0%)	4 (5.6%)	
*PIK3CA* (E545K)	3 (3.3%)	0 (0.0%)	3 (4.2%)	
*PIK3CA* (H1047L)	3 (3.3%)	1 (5.0%)	2 (2.8%)	
*PIK3CA* (E545A)	1 (1.1%)	1 (5.0%)	0 (0.0%)	

^a^Included alterations are companion diagnostic biomarkers for FDA-approved PIK3CA inhibitor therapy

^b^Denominator is total number of samples with missense *ESR1* mutations

^c^Fisher's Exact Test

*ESR1* fusions were detected in 55 (2.1%) BC samples, and they were enriched in metastatic (4.0%) compared to local/regional (1.7%) samples (*P* = 0.002) (Fig. 2A and Supplemental Table 3). In the HR + /HER2- subtype, *ESR1* gene fusion frequency was significantly higher in metastatic (5.3%) compared with local/regional (2.0%) samples (*P* < 0.001) (Supplemental Table 3). Among these events, *CCDC170* was the most frequent *ESR1* fusion partner (n = 37 (77.1%)), with 11 other fusion partners identified (22.9%) (Fig. 3B). *ESR1* amplifications were rare (n = 14 (0.5%)) and were found only in HR + /HER2- (n = 10 (0.6%)) and HER2 + (n = 4 (1.1%)) BC, with no difference in frequency of amplifications between local/regional versus metastatic HR + /HER2- BC samples (Fig. 2A and Supplemental Table 3).

### *Co-alterations in HR* + */HER2- BC*

**Fig. 4:**
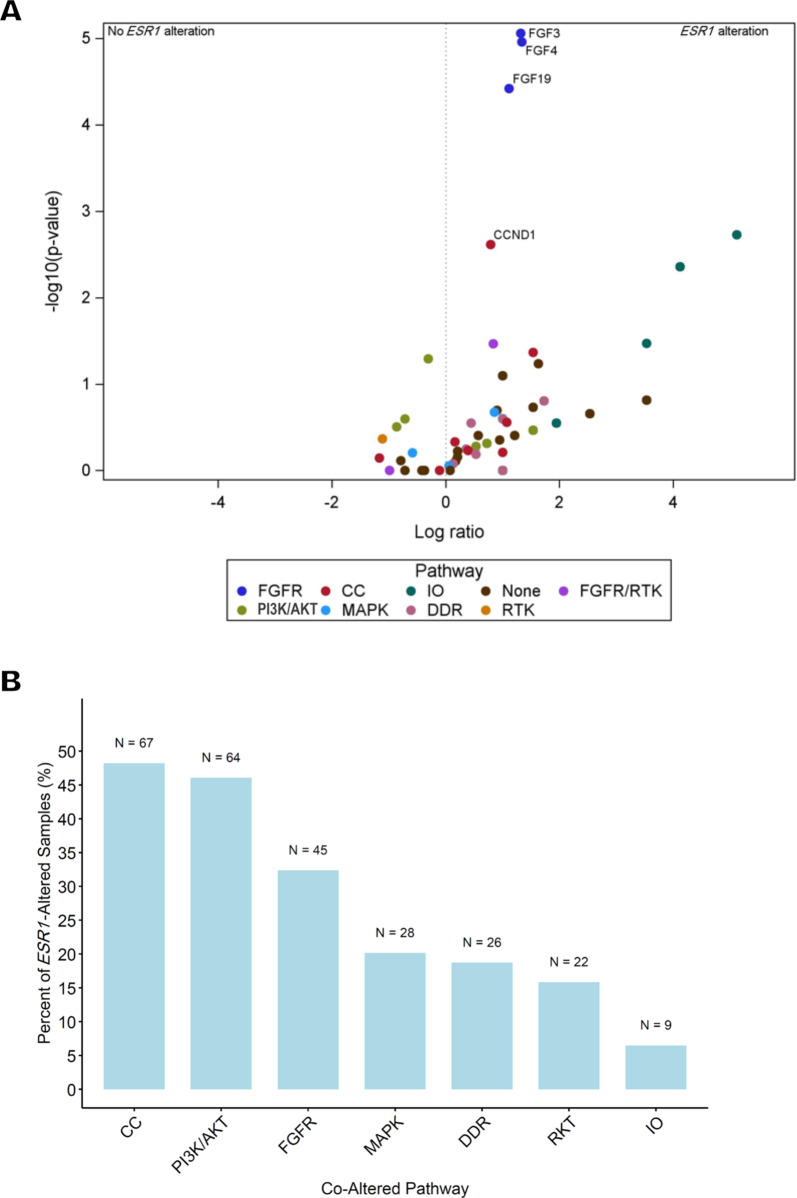
Association of cancer-relevant signaling pathways with *ESR1* alteration status in HR + /HER2- BC samples. A. Co-alterations with *ESR1* in HR + /HER2- BC samples. Magnitude of co-alteration enrichment indicated on the x axis expressed as log2 (percent in *ESR1*-altered / percent *ESR1* wild type). Statistical significance of the association on the y axis expressed as -log10 P value (Fisher’s exact test). Labeled biomarkers represent those that are part of pathways or present in at least 2.5% of *ESR1*-altered or -wild type samples that are significantly associated with *ESR1* alteration status. Significantly enriched co-alterations based on q < 0.05 (Benjamini–Hochberg test). Biomarkers in pathways are grouped by color. FGFR/RTK (Purple) category represents three genes that are included in both cancer-relevant pathways. B. Frequency of co-alteration in at least one gene in seven cancer-relevant pathways in *ESR1*-altered HR + /HER2- BC samples

Endocrine therapy is an important therapeutic modality for HR + /HER2- BC, but both intrinsic and acquired resistance mechanisms can result in inadequate clinical response to modulation of ER signaling. Information regarding the molecular mechanisms driving ET resistance and the genomic landscape of ET-resistant tumors can inform therapy selection to improve patient outcomes. Therefore, we examined the frequency of actionable gene co-alterations with *ESR1* in HR + /HER2- BC. We detected 21 actionable alterations in at least 2.5% of *ESR1-*altered or -non-altered samples, and co-occurring amplification of *FGF3*, *FGF4*, *FGF19*, and *CCND1*, which reside next to each other on the 11q13 chromosome locus, were significantly associated with *ESR1* alterations (q ≤ 0.05 for all comparisons) (Table 2, Fig. 4A). We also identified genes that were not co-altered with *ESR1* in HR + /HER2- BC samples, although no formal statistical comparisons were made: *BRCA1* (n = 24 (1.4%)), *KRAS* (n = 23 (1.3%)), *PRKDC* (n = 13 (0.7%)), *CCNE1* (n = 9 (0.5%)), *CREBBP* (n = 9 (0.5%)), *KDM5C* (n = 9 (0.5%)), *EGFR* (n = 8 (0.5%)), and *NOTCH2* (n = 8 (0.5%)) (Supplemental Table 5). Notably, three of these genes, *KRAS*, *CCNE1*, and *EGFR*, have known roles in ET resistance, and a single alteration in *HRAS*, which can also contribute to ET resistance, was observed in an *ESR1*-wild-type HR + /HER2- BC sample. We found no statistically significant association with *ESR1* alterations among ten other genes known to confer ET resistance (Table 3).

Next, to gain a better understanding of the biological mechanisms driving *ESR1*-altered versus *ESR1*-wild-type disease, we identified HR + /HER2- BC samples with at least one alteration in one of seven cancer-relevant pathways: immuno-oncology (IO), fibroblast growth factor receptor (FGFR), cell cycle (CC), DNA damage repair (DDR), mitogen-activated protein kinase (MAPK*)* signaling, PI3K/AKT signaling, and receptor tyrosine kinase (RTK) signaling (see Supplemental Table 6 for a list of genes included in each of these pathways). From this analysis, we identified a significant association after multiple comparisons correction (q < 0.05) between an *ESR1* alteration and activation of the FGFR signaling pathway (Fig. 4A). Then, we considered all 139 HR + /HER2- BC samples with an *ESR1* alteration and found that co-alterations were most frequent in the cell cycle (n = 67, 48.2%), PI3K/AKT signaling (n = 64, 46.0%), and FGFR signaling (n = 45, 32.4%) pathways (Fig. 4B).

**Fig. 5:**
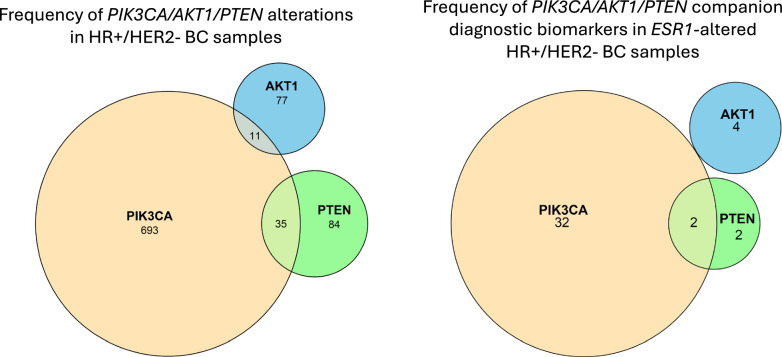
Co-occurrence of *ESR1* and *PIK3CA/AKT1/PTEN* pathway alterations in HR + /HER- BC samples

In addition to *ESR1*, ET resistance can also occur due to dysregulation of the PI3K/AKT pathway, and co-alteration of *ESR1* and PI3K/AKT signaling has also been reported in patients with HR + /HER2- BC [31–33]. Importantly, there are reports of clinical benefit of elacestrant plus alpelisib in patients with co-alteration in *PIK3CA* and *ESR1* [22, 34, 35]. Therefore, we evaluated co-occurring *ESR1* and *PIK3CA/PTEN/AKT1* alterations approved for elacestrant, alpelisib, and capivasertib treatment in HR + /HER2- BC samples. Our data showed that, in 92 HR + /HER2- BC samples with *ESR1* missense mutations, 35 samples (38.0%) had alterations detected in *PIK3CA* and 17 (18.5%) were clinically relevant H1047R/L mutations in the kinase domain (Table 4). There was no difference in the distribution of *PIK3CA* alterations between local/regional and metastatic samples with *ESR1* missense mutations. In HR + /HER2- BC samples, 34 samples (1.9%) had co-occurring *ESR1* and *PIK3CA* alterations, 4 samples (0.2%) had co-occurring *ESR1* and *AKT1* alterations, and 4 samples (0.2%) had co-occurring *ESR1* and *PTEN* alterations associated with FDA-approved therapies (on-label; see Methods) (Fig. 5).

### ESR1 alterations in gynecologic cancer samples

**Fig. 6:**
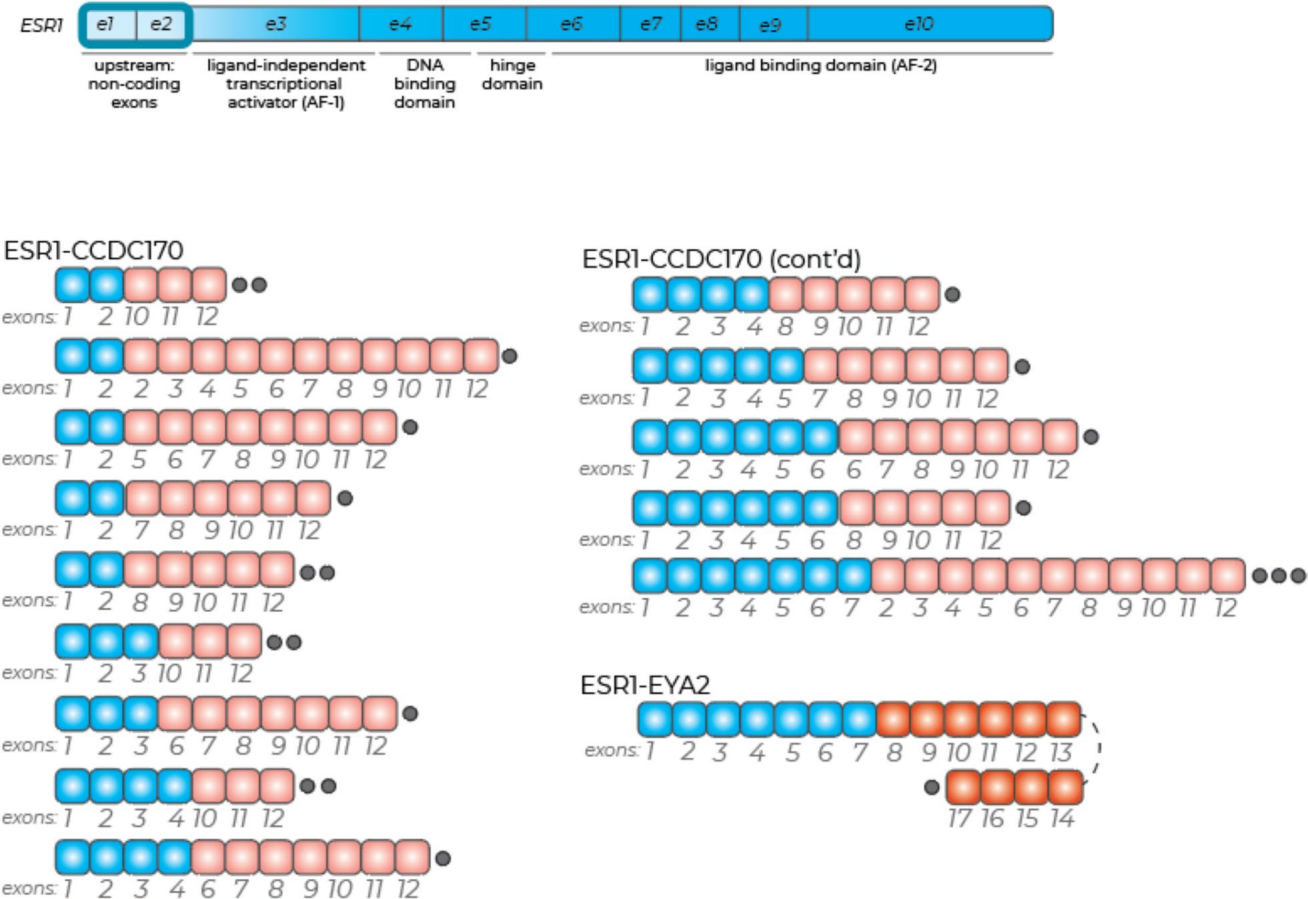
Characterization of *ESR1* fusions in gynecologic cancer samples. Gray dots indicate number of events

Overall, an *ESR1* alteration was detected in 3.4% of gynecologic cancer samples (38 samples), and the frequency of *ESR1* alterations was not significantly different across cancer type, between age groups, or between local/regional versus metastatic site subgroups (Table 1). The distribution of *ESR1* by alteration type also did not differ between local/regional and metastatic sites (Fig. 2A and Supplemental Table 7). Missense mutations were the most common *ESR1* alteration (18 samples (1.6%), with one sample harboring two *ESR1* mutations) and were observed in known hotspots, including Y537C/S (n = 6 (0.05%)), D538G (n = 5 (0.5%)), and L536P (n = 5 (0.5%)) (Fig. 2B and Supplemental Table 8). Among 21 samples (1.9%) with *ESR1* fusions, *CCDC170* was the most common fusion partner (20 of 21 events), and only one other event was an *ESR1-EYA2* fusion (Fig. 6, Supplemental Table 9). Evaluation of 68 biomarkers in all gynecologic cancer samples did not identify any significant associations with the presence or absence of *ESR1* alterations after correcting for multiple comparisons (Supplemental Table 10).

## Discussion

*ESR1* is a known oncogenic driver in breast and, to a lesser extent, endometrial cancers, and ET can be used to inhibit *ESR1* transcriptional and non-genomic activity to achieve therapeutic benefit. Moreover, *ESR1* alterations are a critical mechanism of acquired ET resistance in HR + /HER2- BC and other ER-driven tumor types. In this retrospective study, we observed expected enrichment of *ESR1* alterations in HR + /HER2- metastatic BC tissues at frequencies consistent with previous reports describing acquired resistance alterations in advanced disease, although this study lacked clinical treatment data. We also uncovered pathogenic co-alterations along with *ESR1* which could inform clinical decision-making. Finally, we report what, to our knowledge, is the most comprehensive characterization of *ESR1* alterations in gynecologic cancers to date.

The reported somatic mutation landscapes of primary and metastatic BC have shown that, although metastatic lesions share truncal mutations with the primary tumor, acquired alterations that contribute to therapeutic resistance and disease progression occur under the selective pressure of treatment, resulting in subclonal and/or polyclonal alterations [10, 36–38]. In ET-resistant HR + BC, *ESR1* LBD mutations in solid tissue samples have been reported at frequencies ranging from approximately 10–40% [16, 39–44]. Although our study lacked treatment data, we observed a similar frequency of *ESR1* LBD mutations (22.4%) in metastatic HR + /HER2- BC samples, which have been proposed to arise late and in a subclonal manner, likely the consequence of acquired ET resistance [39]. In comparison, the frequency of *ESR1* LBD mutations in our study in local/regional HR + /HER2- BC samples was only 1.4%, significantly lower than in the metastatic tissues, and consistent with previous reports of *ESR1* alteration frequency in primary BC samples [10, 15, 45]. These observations underscore the need to conduct NGS of metastatic instead of primary BC tissues to detect mutations acquired under the selective pressure of ET, including *ESR1* LBD mutations.

Some studies that have reported relatively higher *ESR1* alteration frequencies tested ctDNA in liquid biopsy samples. Circulating tumor DNA detection of *ESR1* alterations can be more suited for capturing intrapatient mutational heterogeneity, and it can be used for longitudinal sampling, which is relevant given the positive correlation between increased lines of therapy and emergence of *ESR1* resistance mutations [14, 42, 44, 46–49]. Mutational analysis of ctDNA versus matched tumor sample often showed additional *ESR1* mutations beyond the one identified in the tumor sample, suggesting superiority of ctDNA testing in integrating data from different metastatic sites [39]. We also analyzed presence of multiple *ESR1* missense mutations in the same sample and found 4 samples in the HR + /HER2- cohort and 1 sample in the gynecologic cancer cohort with polyclonal events, likely arising from divergent clones. While this represented 4.3% of *ESR1*-mutated HR + /HER2- BC samples in our population, other reports using ctDNA analysis have reported higher frequencies [39]. Moreover, ctDNA monitoring for acquired resistance mutations in *ESR1* is shaping the positioning of oral SERDs in the clinic [50]. A notable example is the SERENA-6 trial, which showed that switching from AI plus CDK4/6 inhibitor therapy to the oral SERD camizestrant plus continued CDK4/6 inhibitor therapy upon detection of circulating mutated *ESR1* significantly improved PFS [51].

We observed a non-random distribution of *ESR1* alterations among distant organs, with the highest prevalence in liver (48 of 151 liver samples (31.8%)) and bone (17 of 53 bone samples (32.1%)). In pairwise comparisons, both liver and bone were significantly enriched in *ESR1* alterations compared with other metastatic sites. Significant enrichment of *ESR1* alterations in liver metastases from patients with BC have been reported previously [10, 12, 52–55], and *ESR1*-altered cases of ER + BC have a higher incidence of liver metastases, suggesting patients with *ESR1*-altered BC may benefit from monitoring for liver lesions [56]. It was also shown that *ESR1*-altered liver metastases had distinct transcriptional programs that may regulate liver metastatic potential as well as a higher frequency of *AGO2* copy number amplifications, which is known to interact with pro-metastatic protein LASP1 [56]. Other studies have reported enrichment of *ESR1* mutations in 20% of bone metastases [12]. One possible explanation for different patterns of organotropism across studies could be the BC subtype composition, as subtype influences organotropic metastasis [57, 58]. It is also possible that our data reflect imbalances in treatment decisions rather than a direct influence of metastatic site on the genetic profile. With the dataset available, it is not possible to distinguish between these possibilities.

Y537C/N/S and D538G have been reported to be the most common *ESR1* LBD mutations [10, 45]. Indeed, the frequencies of these mutations among the HR + /HER2- BC samples that harbored an *ESR1* LBD mutation—43.5% and 39.1%, respectively—were each higher than the combined frequencies of all other *ESR1* LBD alterations (21.7%) in our HR + /HER2- BC cohort. Functional studies and clinical data indicate that, although all LBD mutations are considered activating mutations, their effects on downstream signaling and responsiveness to therapy can vary widely. For example, Y537S and D538G mutations have been shown to promote increased binding of *ESR1* with co-regulators of ER-ligand complexes, and Y537S can more potently enhance hormone-independent transcriptional regulation in BC cells [42, 43]. Importantly, the conformational changes introduced by Y537S and D538G mutations substantially decrease ER binding affinity to some SERMs and SERDs by up to tenfold [45, 59, 60, 61]. Moreover, Y537S-mutant *ESR1* promotes transcription of more genes and more aggressive disease in animal models compared to D538G-mutant *ESR1* [62].

*ESR1* fusion genes are less common than missense mutations, and their biological and therapeutic significance in BC is less well characterized. These fusion constructs almost uniformly lose the *ESR1* LBD, leading to ET resistance [63–65]. Generally, N-terminal *ESR1* sequences lacking the hormone-binding domain fuse to other proteins, where they can act as a promoter trap leading to increased expression of possibly oncogenic proteins/protein truncations [64]. Studies suggest that *ESR1* fusions more frequently occur in ET-resistant, progressive disease, and occur in 2.1% of luminal B subtype BC in the TCGA ER + BC cohort [66]. Similarly, in our cohort, *ESR1* fusions were significantly more frequent in metastatic compared with local/regional BC tissues overall (*P* = 0.002), including in the HR + /HER2- BC subtype (*P* < 0.001).

Utilization of whole transcriptome sequencing with our assay identified 11 rare *ESR1* fusion partners, each of which occurred once. Two of the gene partners, *ARNT2* [67] and *PLEKHG1* [68], have been reported previously. In agreement with previous reports, *CCDC170* was the most common partner gene found among HR + /HER2- BC samples in our study (77.1% of all *ESR1* fusion events in HR + /HER2- BC samples) [69]. One of the most common fusions observed in our study and others involves the first two *ESR1* exons fusing to C-terminal *CCDC170*, generating a truncated CCDC170 protein that has been shown to enhance BC cell growth and decrease tamoxifen sensitivity [66, 70, 71], supporting a role for these fusions in ET resistance. Another study found that exon 2 and exon 8 *ESR1*-*CCDC170* fusion transcripts identify a more aggressive subset of ER-positive breast cancer patients and have prognostic value [72]. Notably, it has been shown that CDK4/6 inhibitors can suppress *ESR1*-fusion-driven growth in some instances, indicating that inhibiting kinases downstream of ER may be an effective therapeutic strategy [64, 73, 74]. However, increased signaling through SRC/HER2/HER3/AKT in breast cancers with *ESR1*-*CCDC170* gene fusions was demonstrated in preclinical models, suggesting additional therapeutic vulnerabilities [71]. Consistently, a case study demonstrated activation of SRC/HER2/HER3/AKT in a BC harboring an *ESR1-CCDC170* fusion, and these cells were sensitive to HER2 (lapatinib) and SRC (dasatinib) inhibition [75].

Examination of co-alterations in HR + /HER2- BC samples at the gene or pathway level revealed associations between *ESR1* alterations and cell cycle regulation, FGFR signaling, and PI3K/AKT signaling. *FGF3*, *FGF4*, *FGF19*, and *CCND1* amplifications were significantly more frequent in *ESR1*-altered compared to *ESR1*-wild-type samples. These genes reside on the 11q13 chromosome locus, which is amplified in approximately 15% of BCs [76, 77]. Evidence suggests that these findings could have important therapeutic implications for patients with *ESR1*-altered tumors. Amplification of *FGF3/4/19* has been associated with responsiveness to sorafenib in patients with advanced hepatocellular carcinoma (HCC) [78, 79], and clinical benefit with RTK inhibitors in FGFR-pathway-altered solid tumors has also been reported [80]. In the MONALEESA-7 trial, greater benefit from the CDK4/6 inhibitor ribociclib was observed in patients with HR + /HER2- BC with *CCND1* amplification [81] and inhibition of CDK4/6 inhibitors has been considered as a therapeutic strategy to overcome endocrine resistance in patients with *PIK3CA-* or *ESR1-*mutant BC [82]. In the MONALEESA-2 trial of first-line ET plus ribociclib, the presence of *FGFR1* amplification was associated with reduced effectiveness of ribociclib. Thus, the observed co-occurrence of *FGFR1* amplification and *ESR1* mutations in a subset of patients suggests that combined inhibition of these alterations will be needed to achieve therapeutic benefit [83].

Besides *ESR1* alterations, the selective pressure of ET has been reported to result in other resistance alterations. Razavi et al. found *ERBB2* gain-of-function mutations and *NF1* loss-of-function mutations to be significantly more common in ET-treated compared to treatment-naïve metastatic BC samples, and that these alterations mostly occurred in *ESR1*-wild-type samples [10]. In the same study, a pathway analysis of treated and untreated metastatic BC samples uncovered hotspot mutations in genes in the MAPK pathway (*ERBB3*, *KRAS*, *HRAS*, *BRAF*, and *MAP2K1*) that were mutually exclusive with *ESR1* alterations in post-treatment samples; these were associated with poor response to AI therapy as well as diminished PFS. Similarly, we observed that *KRAS* and *HRAS* alterations only occurred in *ESR1*-wild-type samples. Combined with the ER-negative phenotype of BC cells with upregulated MAPK signaling [84], these results add further evidence that the MAPK cascade can underly ET resistance and disease progression in BC. Our study as well as that of Razavi’s team [10] also found that *EGFR* focal amplifications were only present in *ESR1*-wild-type samples. In the latter study, these alterations occurred in patients who had received tamoxifen and/or AI therapy, suggesting a possible role in acquired therapy resistance. Thus, genomic profiling of BC at the time of progression on ET could uncover evidence of multiple resistance mechanisms that may be addressable with EGFR or MAPK-pathway inhibitors.

As in HR + BC, *ESR1* alterations in gynecologic cancers can occur in response to ET exposure [27]. *ESR1* LBD mutations have been associated with poor prognosis in women with endometrial cancer [85]. Interestingly, it was reported that a patient with stage IIIC low grade primary peritoneal serous carcinoma with an activating mutation in *ESR1* had clinical benefit with ER-targeted therapy [27]. In our cohort of combined gynecologic cancers, *ESR1* alteration was detected in 3.4% of samples, which is consistent with the frequencies reported for specific gynecologic tumor types in smaller studies. *ESR1* fusions and missense mutations occurred at similar frequencies (1.9% and 1.6%, respectively), and one sample had co-occurring fusion and missense mutation. This suggests that the distribution of *ESR1* alteration types may differ between gynecologic cancer and BC. Furthermore, the potential clinical relevance of this finding is unclear, as the biological characterization of specific *ESR1* alterations in gynecologic tumors is under-reported compared to BC.

Our evaluation of 21 *ESR1* fusions in gynecologic cancer samples showed that, like in HR + /HER2- BC samples, the majority (20 of 21) of events occurred with the partner gene *CCDC170*, and that these commonly involved only the first few *ESR1* exons, likely serving as a promoter trap. Another fusion partner, *EYA2*, was only observed in gynecologic cancer samples. Also similar to HR + /HER2- BC samples, the most common *ESR1* LBD mutations in our gynecologic cancer cohort were Y537C/S (0.5%) and D538G (0.5%). In vitro, the D538G mutant was shown to exert estrogen-independent neomorphic activities [86], suggesting that, like BC, particular LBD mutations may have important implications for therapy selection in *ESR1*-altered gynecologic tumors. Several studies have shown emergence of *ESR1* mutations in endometrial cancer patients on ET, and several trials are underway targeting mutant *ESR1* with oral SERD combination therapies in these patients [87–89]. These findings emphasize the need for further evaluation of *ESR1* mutation status in gynecologic tumors, functional characterization of specific alterations in relevant models, and trials of oral SERDs alone and in combination with CDK4/6 and AKT inhibitors, especially in metastatic endometrial cancer.

Increasing access to genomic profiling has expanded our understanding of the molecular complexity, heterogeneity, and evolution of tumors that drive ET resistance, which is defining the dynamic therapeutic landscape. Although the relevance of *ESR1* alterations for ET resistance is known, the field continues to evaluate how to best treat patients with these molecular alterations. Based on results from the Elacestrant versus Standard Endocrine Therapy for ER + /HER2- Advanced BC (EMERALD) trial, elacestrant is now the first drug approved specifically for patients with *ESR1*-altered HR + /HER2- BC that progressed on prior CDK4/6 inhibitor therapy [22]. There are also novel SERDs currently in late-stage clinical development, including camizestrant, imlunestrant, and giredestrant [21, 90, 91]. In addition, the combination of elacestrant with inhibitors of the PI3K/AKT pathway, including capivasertib, alpelisib, and inavolisib, is being evaluated in multiple clinical trials, such as the ELEVATE trial (NCT05563220). Thus, increasingly, genomic profiling to identify *ESR1* alterations, including the less common gene fusion events, presents an important clinical opportunity to tailor targeted therapy for patients with HR + BC or gynecologic malignancies.

The limitations of this study should be considered when interpreting these findings. First, this was a retrospective study of a database that lacked treatment or outcomes information. Our study also lacked longitudinal samples to inform molecular drivers of disease progression. Finally, some of the alterations evaluated for co-occurrence or mutual exclusivity with an *ESR1* alteration were detected in a small number of samples, which could affect the statistical outcomes. Nevertheless, our work presents important genomic characterization of relatively large cohorts of BC by subtype as well as, to our knowledge, represents the largest study characterizing *ESR1* alterations in gynecologic cancers to date. In addition, the use of whole exome and whole transcriptome profiling enabled a thorough evaluation of the frequency of co-occurring alterations, including companion diagnostic biomarkers currently in clinical use to qualify patients with *ESR1*-altered metastatic BC for therapy with elacestrant.

## Conclusions

Overall, *ESR1* alterations, including missense mutations in the LBD and fusions, were most common in HR + /HER2- BC samples. In this BC subtype and in BC overall, missense mutations and fusions were more common in metastatic biopsy than local/regional samples. In HR + /HER2- BC, cell cycle and FGFR signaling, including amplification of the chromosome locus containing *FGF3*, *FGF4*, *FGF19*, and *CCND1*, were significantly associated with the presence of an *ESR1* alteration. Clinically relevant co-occurring alterations in *ESR1* and the *PI3K*/*AKT*/*PTEN* pathway were detected in 2.3% of HR + /HER2- BC samples. Finally, we characterized the distribution of *ESR1* fusions and missense mutations in a large cohort of gynecologic cancer samples and provide new insights into the nature of these alterations.

## Supplementary Information

Supplementary material 1

## Data Availability

The authors affirm that the data supporting the findings of this study are available within the article and/or its supplementary information files.

## List of Abbreviations

AI: Aromatase inhibitor
BC: Breast cancer
CAP: College of American Pathologists
CC: Cell cycle
CLIA: Clinical Laboratory Improvement Amendments
DDR: DNA damage repair
ER: Estrogen receptor α
ET: Endocrine therapy
FDR: False discovery rate
FGFR: Fibroblast growth factor receptor
HR +: Hormone receptor positive
IRB: Investigational review board
LBD: Ligand-binding domain
MAPK: Mitogen-activated protein kinase
MSI: Microsatellite instability
NOS: Not otherwise specified
OS: Overall survival
RTK: Receptor tyrosine kinase
SERD: Selective estrogen receptor degrader
SERM: Selective estrogen receptor modulator
SNV: Single-nucleotide variant
TMB: Tumor mutational burden
TNBC: Triple-negative breast cancer
