# Supplementary material for: Characterization of ESR1 alterations in patients with breast and gynecologic cancers
Source: Breast Cancer Res. 2026 Jan 19;28:40. doi: 10.1186/s13058-025-02217-0 (PMC12879367; doi:10.1186/s13058-025-02217-0)
Supplement: Supplementary file 1 — Supplementary material 1 [file 13058_2025_2217_MOESM1_ESM.docx]

**SUPPLEMENTAL TABLES**

**Supplemental Table 1. Specimen site in BC patients, by BC subtype and *ESR1* alteration status.**

|  | **HER2+ (N=369)** | | | **HR+/HER2- (N=1748)** | | | **TNBC (N=387)** | | | **NOS (N=70)** | | |
| --- | --- | --- | --- | --- | --- | --- | --- | --- | --- | --- | --- | --- |
| **Specimen Site^a,b^** | ***ESR1* Alt** | **No *ESR1* Alt** | **P-value^c^** | ***ESR1* Alt** | **No *ESR1* Alt** | **P-value^c^** | ***ESR1* Alt** | **No *ESR1* Alt** | **P-value^c^** | ***ESR1* Alt** | **No *ESR1* Alt n (%)** | **P-value^c^** |
| Overall | 14 (3.8%) | 355 (96.2%) |  | 139 (8.0%) | 1609 (92.0%) |  | 1 (0.3%) | 386 (99.7%) |  | 5 (7.1%) | 65 (92.9%) |  |
| Local/Regional | 10 (2.7%) | 309 (83.7%) | 0.11 | 53 (3.0%) | 1374 (78.6%) | <0.001 | 1 (0.3%) | 299 (77.3%) | 1.00 | 1 (1.4%) | 55 (78.6%) | 0.005 |
| Metastatic | 4 (1.1%) | 46 (12.5%) |  | 86 (4.9%) | 235 (13.4%) |  | 0 (0.0%) | 87 (22.5%) |  | 4 (5.7%) | 10 (14.3%) |  |
| Bone | 0 (0%) | 4 (1.1%) |  | 17 (1.0%) | 26 (1.5%) |  | 0 (0%) | 0 (0%) |  | 0 (0%) | 2 (2.9%) |  |
| Brain | 3 (0.8%) | 8 (2.2%) |  | 0 (0%) | 0 (0%) |  | 0 (0%) | 5 (1.3%) |  | 0 (0%) | 0 (0%) |  |
| Chest Wall | 0 (0%) | 5 (1.4%) |  | 3 (0.2%) | 21 (1.2%) |  | 0 (0%) | 10 (2.6%) |  | 1 (1.4%) | 2 (2.9%) |  |
| Liver | 0 (0%) | 7 (1.9%) |  | 46 (2.6%) | 79 (4.5%) |  | 0 (0%) | 16 (4.1%) |  | 2 (2.9%) | 1 (1.4%) |  |
| Lung | 1 (0.3%) | 8 (2.2%) |  | 1 (0.1%) | 17 (1.0%) |  | 0 (0%) | 9 (2.3%) |  | 0 (0%) | 0 (0%) |  |
| Other | 0 (0%) | 7 (1.9%) |  | 14 (0.8%) | 68 (3.9%) |  | 0 (0%) | 24 (6.2%) |  | 1 (1.4%) | 5 (7.1%) |  |
| Skin | 0 (0%) | 7 (1.9%) |  | 5 (0.3%) | 24 (1.4%) |  | 0 (0%) | 18 (4.7%) |  | 0 (0%) | 0 (0%) |  |
| Soft Tissue | 0 (0%) | 0 (0%) |  | 0 (0%) | 0 (0%) |  | 0 (0%) | 5 (1.3%) |  | 0 (0%) | 0 (0%) |  |

^a^Primary specimen site defined as breast, lymph node, and axilla; Metastatic defined as any other specimen site
^b^Denominator is number of samples within BC subtype; Metastatic sites that occur in <5% of metastatic samples and less than 2 samples are categorized as 'Other'
^c^P-values were derived from Chi-Square and Fisher's Exact Tests for comparison of primary vs. metastatic samples

**Supplemental Table 2. Pairwise comparison of *ESR1* alteration frequency by metastatic site in BC samples.**

| **Pairwise Comparison (n *ESR1* alts/n total^a^)** | |  |
| --- | --- | --- |
| **Metastatic Site A** | **Metastatic Site B** | **Adjusted P-value^b^** |
| Liver (48/151) | Skin (5/55) | 0.011 |
| Liver (48/151) | Lung (2/37) | 0.013 |
| Bone (17/53) | Lung (2/37) | 0.025 |
| Bone (17/53) | Skin (5/55) | 0.033 |
| Liver (48/151) | Chest Wall (4/41) | 0.05 |
| Bone (17/53) | Chest Wall (4/41) | 0.11 |
| Liver (48/151) | Brain (3/24) | 0.59 |
| Bone (17/53) | Brain (3/24) | 0.77 |
| Liver (48/151) | Soft Tissue (3/19) | 1.00 |
| Bone (17/53) | Soft Tissue (3/19) | 1.00 |
| Liver (48/151) | Bone (17/53) | 1.00 |

^a^ Within each metastatic site

^b^ Chi-square test adjusted for multiple testing

**Supplemental Table 3. *ESR1* alterations in BC, overall and by specimen site**

| **BC Subtype** |  | **All Samples n (%^a^)** | **Local/Regional n (%^a^)** | **Metastatic n (%^a^)** | **P-value^b^** |
| --- | --- | --- | --- | --- | --- |
| Overall | All Samples | 2574 | 2102 (81.7%) | 472 (18.3%) |  |
|  | Any *ESR1* Alteration | 159 (6.2%) | 65 (3.1%) | 94 (19.9%) | <0.001 |
|  | *ESR1* Missense | 99 (3.8%) | 23 (1.1%) | 76 (16.1%) | <0.001 |
|  | *ESR1* Amplification | 14 (0.5%) | 11 (0.5%) | 3 (0.6%) | 0.73 |
|  | *ESR1* Fusion | 55 (2.1%) | 36 (1.7%) | 19 (4.0%) | 0.002 |
|  | *ESR1* mutation/fusion | 4 (0.2%) | 0 (0.0%) | 4 (0.8%) | 0.001 |
| HER2+ | All Samples | 369 | 319 (12.4%) | 50 (1.9%) |  |
|  | Any *ESR1* Alteration | 14 (3.8%) | 10 (3.1%) | 4 (8.0%) |  |
|  | *ESR1* Missense | 2 (0.5%) | 1 (0.3%) | 1 (2.0%) |  |
|  | *ESR1* Amplification | 4 (1.1%) | 2 (0.6%) | 2 (4.0%) |  |
|  | *ESR1* Fusion | 8 (2.2%) | 7 (2.2%) | 1 (2.0%) |  |
|  | *ESR1* mutation/fusion | 0 (0.0%) | 0 (0.0%) | 0 (0.0%) |  |
| HR+/HER2- | All Samples | 1748 | 1427 (55.4%) | 321 (12.5%) |  |
|  | Any *ESR1* Alteration | 139 (8.0%) | 53 (3.7%) | 86 (26.8%) | <0.001 |
|  | *ESR1* Missense | 92 (5.3%) | 20 (1.4%) | 72 (22.4%) | <0.001 |
|  | *ESR1* Amplification | 10 (0.6%) | 9 (0.6%) | 1 (0.3%) | 0.70 |
|  | *ESR1* Fusion | 46 (2.6%) | 29 (2.0%) | 17 (5.3%) | <0.001 |
|  | *ESR1* mutation/fusion | 4 (0.2%) | 0 (0.0%) | 4 (1.2%) | 0.001 |
| TNBC | All Samples | 387 | 300 (11.7%) | 87 (3.4%) |  |
|  | Any *ESR1* Alteration | 1 (0.3%) | 1 (0.3%) | 0 (0%) |  |
|  | *ESR1* Missense | 1 (0.3%) | 1 (0.3%) | 0 (0%) |  |
|  | *ESR1* Amplification | 0 (0.0%) | 0 (0.0%) | 0 (0.0%) |  |
|  | *ESR1* Fusion | 0 (0.0%) | 0 (0%) | 0 (0%) |  |
|  | *ESR1* mutation/fusion | 0 (0.0%) | 0 (0.0%) | 0 (0.0%) |  |
| NOS | All Samples | 70 | 56 (2.2%) | 14 (0.5%) |  |
|  | Any *ESR1* Alteration | 5 (7.1%) | 1 (1.8%) | 4 (28.6%) |  |
|  | *ESR1* Missense | 4 (5.7%) | 1 (1.8%) | 3 (21.4%) |  |
|  | *ESR1* Amplification | 0 (0.0%) | 0 (0.0%) | 0 (0.0%) |  |
|  | *ESR1* Fusion | 1 (1.4%) | 0 (0%) | 1 (7.1%) |  |
|  | *ESR1* mutation/fusion | 0 (0.0%) | 0 (0.0%) | 0 (0.0%) |  |

^a^ Column % within BC subtype

^b^ Chi-square Test or Fisher's Exact Test

**Supplemental Table 4. Missense variant allele frequency in *ESR1*-altered HR+/HER2- BC patients.**

| **Specimen Site** | **N** | **Mean VAF (SD)** | **Median VAF** | **Q1, Q3, Max** | **P-value^a^** |
| --- | --- | --- | --- | --- | --- |
| Local/Regional | 20 | 17.1 (10.6) | 15 | 10, 26, 38 | 0.019 |
| Metastatic | 72 | 26.1 (15.9) | 23 | 15, 33, 82 |  |

^a^ Wilcoxon Rank-Sum Test

**Supplemental Table 5. Potentially actionable alterations that occurred only in *ESR1*-wild-type HR+/HER2- BC samples.^a^**

| **Co-altered Biomarker** | **Overall (N=1748)** | ***ESR1* alteration (N=139)** | **No *ESR1* alteration (N=1609)** |
| --- | --- | --- | --- |
| KRAS | 23 (1.3%) | 0 (0.0%) | 23 (1.4%) |
| BRCA1 | 24 (1.4%) | 0 (0.0%) | 24 (1.5%) |
| PRKDC | 13 (0.7%) | 0 (0.0%) | 13 (0.8%) |
| CCNE1 | 9 (0.5%) | 0 (0.0%) | 9 (0.6%) |
| CREBBP | 9 (0.5%) | 0 (0.0%) | 9 (0.6%) |
| KDM5C | 9 (0.5%) | 0 (0.0%) | 9 (0.6%) |
| EGFR | 8 (0.5%) | 0 (0.0%) | 8 (0.5%) |
| NOTCH2 | 8 (0.5%) | 0 (0.0%) | 8 (0.5%) |
| SETD2 | 7 (0.4%) | 0 (0.0%) | 7 (0.4%) |
| GNAS | 6 (0.3%) | 0 (0.0%) | 6 (0.4%) |
| PBRM1 | 6 (0.3%) | 0 (0.0%) | 6 (0.4%) |
| SMARCA4 | 6 (0.3%) | 0 (0.0%) | 6 (0.4%) |
| STAG2 | 6 (0.3%) | 0 (0.0%) | 6 (0.4%) |
| BAP1 | 5 (0.3%) | 0 (0.0%) | 5 (0.3%) |
| MAPK1 | 5 (0.3%) | 0 (0.0%) | 5 (0.3%) |

^a^ Genes detected in at least 5 samples are shown.

**Supplemental Table 6. List of genes included in the seven cancer-relevant pathways.**

| **PI3K/AKT Pathway** | **MAPK Pathway** | **DDR Pathway** | **Immuno-oncology Pathway** | **Cell cycle Pathway** | **FGFR Pathway** | **RTK Pathway** |
| --- | --- | --- | --- | --- | --- | --- |
| PIK3CA | KRAS | ARID1A | POLE | CCND1 | FGFR1 | ALK |
| AKT1 | NRAS | ATM | POLD1 | CCND2 | FGFR2 | EGFR |
| AKT2 | HRAS | ATR | PMS2 | CCND3 | FGFR3 | ERBB2 |
| AKT3 | BRAF | ATRX | PMS1 | CDK4 | FGFR4 | FGFR1 |
| PIK3CB | MAP2K1 | BAP1 | PDCD1LG2 | CDK6 | FGF3 | FGFR2 |
| MTOR | MAP2K7 | BARD1 | PBRM1 | RB1 | FGF4 | FGFR3 |
| PTEN | MAP2K2 | BLM | MSH6 | E2F1 | FGF6 | KIT |
| RICTOR | MAP2K4 | BRCA1 | MSH3 | E2F3 | FGF19 | MET |
| RPTOR | MAP3K1 | BRCA2 | MSH2 | SMAD2 | FGF23 | PDGFRA |
| PIK3CD | MAP4K2 | BRIP1 | MLH1 | SMAD3 |  | PDGFRB |
| TSC1 | MAP4K3 | CDK12 | CTLA4 | SMAD4 |  | RET |
| TSC2 | MAP3K8 | CHEK1 | CD274 | CDKN2A |  | ROS1 |
| STK11 | RASGRF1 | CHEK2 | ARID2 | CDKN2B |  |  |
|  | NF1 | EPCAM | MSI-High | CDKN2C |  |  |
|  | RAF1 | ERCC1 | TMB-High (≥10 mut/Mb) | CDKN1B |  |  |
|  | NF2 | ERCC2 |  | CDKN1A |  |  |
|  | RASA1 | ERCC3 |  | CCNE1 |  |  |
|  | ARAF | ERCC4 |  | CDC6 |  |  |
|  |  | ERCC5 |  | CDK1 |  |  |
|  |  | FANCA |  | TP53 |  |  |
|  |  | FANCC |  | CREBBP |  |  |
|  |  | FANCD2 |  | EP300 |  |  |
|  |  | FANCE |  | PRKDC |  |  |
|  |  | FANCF |  | MDM2 |  |  |
|  |  | FANCG |  |  |  |  |
|  |  | FANCI |  |  |  |  |
|  |  | FANCL |  |  |  |  |
|  |  | FANCM |  |  |  |  |
|  |  | MRE11A |  |  |  |  |
|  |  | MUTYH |  |  |  |  |
|  |  | NBN |  |  |  |  |
|  |  | PALB2 |  |  |  |  |
|  |  | PPP2R2A |  |  |  |  |
|  |  | RAD21 |  |  |  |  |
|  |  | RAD50 |  |  |  |  |
|  |  | RAD51 |  |  |  |  |
|  |  | RAD51B |  |  |  |  |
|  |  | RAD51C |  |  |  |  |
|  |  | RAD51D |  |  |  |  |
|  |  | RAD52 |  |  |  |  |
|  |  | RAD54L |  |  |  |  |
|  |  | XRCC1 |  |  |  |  |
|  |  | XRCC2 |  |  |  |  |
|  |  | XRCC3 |  |  |  |  |

**Supplemental Table 7. *ESR1* alterations in gynecologic cancer patients, overall and by specimen site**

|  | **All Samples n (%)** | **Local/Regional n (%^a^)** | **Metastatic n (%^a^)** | **Undefined^b^ n (%^a^)** | **p-value^c^** |
| --- | --- | --- | --- | --- | --- |
| All Samples | 1110 | 572 (51.5%) | 453 (40.8%) | 85 (7.7%) |  |
| Any *ESR1* Alteration | 38 (3.4%) | 19 (3.3%) | 15 (3.3%) | 4 (4.7%) | 0.80 |
| *ESR1* Missense | 18 (1.6%) | 8 (1.4%) | 9 (2.0%) | 1 (1.2%) | 0.72 |
| *ESR1* Amplification | 1 (0.1%) | 0 (0.0%) | 1 (0.2%) | 0 (0.0%) | 0.48 |
| *ESR1* Fusion | 21 (1.9%) | 11 (1.9%) | 6 (1.3%) | 4 (4.7%) | 0.11 |
| *ESR1* mutation/fusion | 1 (0.1%) | 0 (0.0%) | 0 (0.0%) | 1 (1.2%) | 0.08 |

^a^ Percent within specimen site

^b^ Undefined refers to tumors that could not be categorized to either local/regional or metastatic.

^c^ Chi-square Test or Fisher's Exact Test

**Supplemental Table 8. Distribution of *ESR1* mutations in gynecologic cancer samples, overall and by specimen site.**

| ***ESR1* mutation** | **All Samples^a^ (N=1110) n (%)** | **Local/Regional (N=572) n (%)** | **Metastatic (N=453) n (%)** | **Undefined^b^ (N=85) n (%)** |
| --- | --- | --- | --- | --- |
| Amplification | 1 (0.1%) | 0 (0.0%) | 1 (0.2%) | 0 (0.0%) |
| D538G | 5 (0.5%) | 2 (0.3%) | 2 (0.4%) | 1 (1.2%) |
| K303M | 1 (0.1%) | 0 (0.0%) | 1 (0.2%) | 0 (0.0%) |
| L536H | 1 (0.1%) | 0 (0.0%) | 1 (0.2%) | 0 (0.0%) |
| L536P | 5 (0.5%) | 4 (0.7%) | 1 (0.2%) | 0 (0.0%) |
| L536R | 1 (0.1%) | 1 (0.2%) | 0 (0.0%) | 0 (0.0%) |
| Y537# | 6 (0.5%) | 2 (0.3%) | 4 (0.9%) | 0 (0.0%) |
| Y537C | 2 (0.2%) | 1 (0.2%) | 1 (0.2%) | 0 (0.0%) |
| Y537S | 4 (0.4%) | 1 (0.2%) | 3 (0.7%) | 0 (0.0%) |

^a^Denominator is total number of gynecologic samples.

^b^ Undefined refers to tumors that could not be categorized to either local/regional or metastatic.

Note: One sample had two *ESR1* mutations (D538G & L536P).

**Supplemental Table 9. Distribution of *ESR1* fusions in gynecologic cancer patients, overall and by specimen site.**

| ***ESR1* fusion** | **All Samples^a^ (N=1110) n (%)** | **Local/Regional (N=572) n (%)** | **Metastatic (N=453) n (%)** | **Undefined^b^ (N=85) n (%)** |
| --- | --- | --- | --- | --- |
| ESR1/CCDC170 | 20 (1.8%) | 11 (1.9%) | 5 (1.1%) | 4 (4.7%) |
| ESR1/EYA2 | 1 (0.1%) | 0 (0.0%) | 1 (0.2%) | 0 (0.0%) |

^a^Denominator is total number of gynecologic cancer samples.

^b^Undefined refers to tumors that could not be categorized to either local/regional or metastatic.

**Supplemental Table 10. Co-occurrence of potentially actionable alterations in gynecologic cancer samples, overall and by *ESR1* alteration status.**

| **Co-altered Biomarker** | **Overall (N=1110)** | ***ESR1* alteration (N=38)** | **No *ESR1* alteration (N=1072)** | **q-value^a^** |
| --- | --- | --- | --- | --- |
| FGF3 | 11 (1.0%) | 3 (7.9%) | 8 (0.7%) | 0.31 |
| FGF19 | 14 (1.3%) | 3 (7.9%) | 11 (1.0%) | 0.31 |
| MAP2K4 | 6 (0.5%) | 2 (5.3%) | 4 (0.4%) | 0.31 |
| CDK1 | 1 (0.1%) | 1 (2.6%) | 0 (0.0%) | 0.31 |
| RARA | 1 (0.1%) | 1 (2.6%) | 0 (0.0%) | 0.31 |
| ERCC5 | 9 (0.8%) | 2 (5.3%) | 7 (0.7%) | 0.31 |
| FGF4 | 10 (0.9%) | 2 (5.3%) | 8 (0.7%) | 0.31 |
| POLE | 40 (3.6%) | 4 (10.5%) | 36 (3.4%) | 0.31 |
| PIK3CA | 310 (27.9%) | 16 (42.1%) | 294 (27.4%) | 0.31 |
| ARAF | 2 (0.2%) | 1 (2.6%) | 1 (0.1%) | 0.31 |
| BARD1 | 2 (0.2%) | 1 (2.6%) | 1 (0.1%) | 0.31 |
| ERCC4 | 2 (0.2%) | 1 (2.6%) | 1 (0.1%) | 0.31 |
| FANCC | 2 (0.2%) | 1 (2.6%) | 1 (0.1%) | 0.31 |
| MYCL | 2 (0.2%) | 1 (2.6%) | 1 (0.1%) | 0.31 |
| TRAF3 | 2 (0.2%) | 1 (2.6%) | 1 (0.1%) | 0.31 |
| CCND1 | 30 (2.7%) | 3 (7.9%) | 27 (2.5%) | 0.34 |
| ERBB3 | 15 (1.4%) | 2 (5.3%) | 13 (1.2%) | 0.34 |
| STAG2 | 15 (1.4%) | 2 (5.3%) | 13 (1.2%) | 0.34 |
| PIK3R2 | 3 (0.3%) | 1 (2.6%) | 2 (0.2%) | 0.34 |
| PTEN | 307 (27.7%) | 15 (39.5%) | 292 (27.2%) | 0.34 |
| MLH1 | 17 (1.5%) | 2 (5.3%) | 15 (1.4%) | 0.34 |
| APC | 35 (3.2%) | 3 (7.9%) | 32 (3.0%) | 0.34 |
| SETD2 | 18 (1.6%) | 2 (5.3%) | 16 (1.5%) | 0.34 |
| CBL | 4 (0.4%) | 1 (2.6%) | 3 (0.3%) | 0.34 |
| CDK2 | 4 (0.4%) | 1 (2.6%) | 3 (0.3%) | 0.34 |
| MAP2K1 | 4 (0.4%) | 1 (2.6%) | 3 (0.3%) | 0.34 |
| MSH6 | 39 (3.5%) | 3 (7.9%) | 36 (3.4%) | 0.35 |
| MTOR | 20 (1.8%) | 2 (5.3%) | 18 (1.7%) | 0.35 |
| RASA1 | 20 (1.8%) | 2 (5.3%) | 18 (1.7%) | 0.35 |
| KRAS | 157 (14.1%) | 2 (5.3%) | 155 (14.5%) | 0.35 |
| XPO1 | 5 (0.5%) | 1 (2.6%) | 4 (0.4%) | 0.35 |
| ERBB2 | 45 (4.1%) | 3 (7.9%) | 42 (3.9%) | 0.38 |
| ERRFI1 | 6 (0.5%) | 1 (2.6%) | 5 (0.5%) | 0.38 |
| TSC2 | 6 (0.5%) | 1 (2.6%) | 5 (0.5%) | 0.38 |
| SMARCA4 | 25 (2.3%) | 2 (5.3%) | 23 (2.1%) | 0.41 |
| FGFR2 | 52 (4.7%) | 3 (7.9%) | 49 (4.6%) | 0.44 |
| ATM | 51 (4.6%) | 3 (7.9%) | 48 (4.5%) | 0.44 |
| PIK3R1 | 109 (9.8%) | 6 (15.8%) | 103 (9.6%) | 0.44 |
| ATRX | 29 (2.6%) | 2 (5.3%) | 27 (2.5%) | 0.44 |
| MAP3K1 | 29 (2.6%) | 2 (5.3%) | 27 (2.5%) | 0.44 |
| CDKN1B | 9 (0.8%) | 1 (2.6%) | 8 (0.7%) | 0.44 |
| FANCA | 9 (0.8%) | 1 (2.6%) | 8 (0.7%) | 0.44 |
| MYC | 31 (2.8%) | 2 (5.3%) | 29 (2.7%) | 0.44 |
| BLM | 10 (0.9%) | 1 (2.6%) | 9 (0.8%) | 0.44 |
| SLX4 | 10 (0.9%) | 1 (2.6%) | 9 (0.8%) | 0.44 |
| ASXL1 | 32 (2.9%) | 2 (5.3%) | 30 (2.8%) | 0.44 |
| FBXW7 | 72 (6.5%) | 4 (10.5%) | 68 (6.3%) | 0.44 |
| MLH3 | 11 (1.0%) | 1 (2.6%) | 10 (0.9%) | 0.45 |
| TP53 | 543 (48.9%) | 22 (57.9%) | 521 (48.6%) | 0.45 |
| PBRM1 | 13 (1.2%) | 1 (2.6%) | 12 (1.1%) | 0.50 |
| TET2 | 14 (1.3%) | 1 (2.6%) | 13 (1.2%) | 0.52 |
| FANCM | 16 (1.4%) | 1 (2.6%) | 15 (1.4%) | 0.56 |
| CTNNB1 | 139 (12.5%) | 6 (15.8%) | 133 (12.4%) | 0.59 |
| NRAS | 19 (1.7%) | 1 (2.6%) | 18 (1.7%) | 0.61 |
| RAD50 | 21 (1.9%) | 1 (2.6%) | 20 (1.9%) | 0.65 |
| ATR | 24 (2.2%) | 1 (2.6%) | 23 (2.1%) | 0.68 |
| CDK12 | 24 (2.2%) | 1 (2.6%) | 23 (2.1%) | 0.68 |
| PRKDC | 34 (3.1%) | 0 (0.0%) | 34 (3.2%) | 0.73 |
| CCNE1 | 38 (3.4%) | 0 (0.0%) | 38 (3.5%) | 0.73 |
| RB1 | 43 (3.9%) | 2 (5.3%) | 41 (3.8%) | 0.74 |
| RNF43 | 48 (4.3%) | 2 (5.3%) | 46 (4.3%) | 0.76 |
| BRCA2 | 65 (5.9%) | 1 (2.6%) | 64 (6.0%) | 0.79 |
| ARID1A | 272 (24.5%) | 9 (23.7%) | 263 (24.5%) | 1.00 |
| BRCA1 | 56 (5.0%) | 1 (2.6%) | 55 (5.1%) | 1.00 |
| NF1 | 53 (4.8%) | 1 (2.6%) | 52 (4.9%) | 1.00 |
| AKT1 | 34 (3.1%) | 1 (2.6%) | 33 (3.1%) | 1.00 |
| MSH3 | 34 (3.1%) | 1 (2.6%) | 33 (3.1%) | 1.00 |
| CREBBP | 30 (2.7%) | 1 (2.6%) | 29 (2.7%) | 1.00 |

^a^ Fisher's Exact Test adjusted using Benjamini-Hochberg FDR q-value
Note: Genes included if present in at least 2.5% of *ESR1*-altered or non-altered samples.
Sorted by descending significance of co-alteration.
